# Prognostic factors for permanent neurological dysfunction after total aortic arch replacement with regional cerebral oxygen saturation monitoring

**DOI:** 10.1002/brb3.1309

**Published:** 2019-05-29

**Authors:** Ying Yu, Yi Lyu, Lin Jin, Liying Xu, Huilin Wang, Yan Hu, Yun Ren, Kefang Guo

**Affiliations:** ^1^ Department of Anesthesiology Zhongshan Hospital, Fudan University Shanghai China; ^2^ Department of Anesthesiology Yunnan Baoshan Anli Hospital Baoshan China

**Keywords:** deep hypothermic circulatory arrest, permanent neurological dysfunction, prognostic factors, regional cerebral oxygen saturation monitoring, total aortic arch replacement

## Abstract

**Objective:**

To explore the prognostic factors for permanent neurological dysfunction (PND) after total aortic arch replacement with regional cerebral oxygen saturation (rSO_2_) monitoring.

**Methods:**

This retrospective study enrolled 98 type A aortic dissection aneurysm patients who underwent emergency total aortic arch replacement combined with deep hypothermic circulatory arrest and right axillary artery selective antegrade cerebral perfusion (SACP). Data such as age, gender, body mass index, preoperative coexisting disease, laboratory test results, intraoperative critical operation duration, and intraoperative rSO_2_ were collected, and the neurological prognoses in the hospital were recorded and grouped by severity. Multiple logistic regression analysis was performed on the statistically significant differences between the groups to screen the predictors of postoperative neurological complications in these patients.

**Results:**

Forty‐two patients had postoperative neurological complications, among which there were 29 cases (29.6%) of transient neurological dysfunction, and 13 cases (13.3%) of PND. Multiple logistic regression results showed that advanced age, preoperative low platelet count, prolonged hemostasis time and lowest relative rSO_2_ to baseline (ΔrSO_2_min) in each time period were risk factors for postoperative PND. The ROC curve measurement showed that the optimal cut‐off value of ΔrSO_2_min was 79.7%, and the area under the curve was 0.708 (95% confidence interval = 0.557–0.858), *p* = 0.016; the optimal cut‐off value of ΔrSO_2_min in SACP was 81.6%, and the area under the curve was 0.720 (95% confidence interval = 0.570–0.870), *p* = 0.011; the optimal cut‐off value of ΔrSO_2_min in cardiopulmonary bypass (CPB) was 80.8%, and the area under the curve was 0.697 (95% confidence interval = 0.554–0.840), *p* = 0.023.

**Conclusion:**

Intraoperative ΔrSO_2_min that is lower than the basal level of about 80%, advanced age, preoperative low platelet count, and prolonged hemostasis time are predictors of PND after total aortic arch replacement.

## 1 INTRODUCTION

Total aortic arch replacement with a long duration and complicated operation has a great chance of postoperative complications of various systems. Factors such as ischemia, hypoxia, reperfusion injury, and internal environment disturbance caused by deep hypothermic circulatory arrest (DHCA) during surgery may lead severe damage to central nervous system. Disturbance of consciousness, paresthesia, physical activity disorder, and cognitive impairment are common injuries, and these permanent neurological complications will decrease the quality of life significantly (Minakawa et al., 2010). These difficult complications always confuse clinicians.

Currently, the significance of brain function monitoring during the cardiovascular suegeries is still unclear (Serraino & Murphy, 2017; Zheng et al., 2013). Near‐infrared spectroscopy (NIRS) is a technique used for percutaneous monitoring of regional cerebral oxygen saturation (rSO_2_), which can reflect the oxygen supply and demand balance of brain tissue in a noninvasive, real‐time and continuous manner (Jobsis, 1977); it has been applied to monitor perioperative brain function. Some studies have suggested that rSO_2_ is associated with the prognosis such as postoperative mortality (Hansen et al., 2013), stroke (Tournay‐Jette et al., 2011), delirium (Khazi, Al‐Safadi, Al, & Aljassim, 2018), cognitive dysfunction (Kakihana et al., 2012), and extended hospital stay (Goldman, Sutter, Ferdinand, & Trace, 2004) of some cardiac surgeries such as coronary artery bypass surgery or valve replacement surgery. However, the range of optimal rSO_2_ is still lacking.

In this study, Stanford type A aortic dissection patients undergoing emergency total aortic arch replacement were studied, whose correlation between various factors of perioperative surgery including intraoperative rSO_2_ changes and postoperative neurological complications were analyzed to search for correctable risk factors and to verify the predictive value of rSO_2_ for neurological prognosis in such surgeries, to provide more references for the clinical practice of predicting and improving the neurological prognosis after total arch replacement.

## 2 MATERIALS AND METHODS

### 2.1 Study design and participants

This is a retrospective study of 98 patients who underwent total aortic arch replacement in our hospital between 2013 and 2015. Inclusion criteria: (a) acute Stanford type A aortic dissection; (b) aged from 20 to 74, male or female; (c) emergency surgery; and (d) intraoperative monitoring of patients with INVOS 4100 NIRS monitor (Somanetics, USA) for bilateral rSO_2_. Exclusion criteria: (a) noninitial cardiac surgery or preoperative aortic stent implanted; (b) preoperative neurological injury, based on symptoms, signs, and imaging data; (c) preoperative renal insufficiency with dialysis indications; and (d) uncooperative mental abnormalities.

### 2.2 Anesthesia and operation protocol

After heparinization, the patients were underwent unilateral femoral artery and right axillary artery cannula, followed by cardiopulmonary bypass (CPB) and total body hypothermia. Cold blood cardioplegia with elevated potassium concentration was infused directly into both the coronary arteries. After cooling down to standard (nasal pharyngeal temperature 18–20°C, bladder temperature 22–25°C), DHCA was started, and unilateral selective antegrade cerebral perfusion (SACP) was performed through the right axillary artery. Triple‐branched stent graft implantation technique was applied in all of the surgeries. The lower aortic circulation was restored after anastomosing the opening and the stent proximal end of the descending aortic. Branches of artificial and left subclavian artery, left common carotid artery were anastomosed successively, and distal circulation was restored and began rewarming. Innominate artery and branch of artificial graft were anastomosed. The CPB was routinely terminated, and hemostasis and chest closure were performed.

Anesthesia was performed using intravenous anesthesia combined with inhalation anesthesia, including propofol, sevoflurane, sufentanil, dexmedetomidine and rocuronium. From the start of CPB, ice packs were placed around the head of patients until the start of rewarming. During the hemostasis period, the systolic blood pressure was controlled at 60–90 mmHg (1 mmHg = 0.133 kPa), and Hb ≥ 8.0 g/L and HCT ≥ 24% were maintained. The NIRS monitor was used to monitor the bilateral forehead rSO_2_. The patients were tranferred to ICU with trachea cannula.

### 2.3 Data collection and definition

Patients’ information including age, sex, body mass index, preoperative comorbidities and laboratory findings, rSO_2_ at various key steps during surgery, postoperative mortality and neurological complications were collected. All data recordings were cut‐off at discharge.

The prognosis of the nervous system was recorded in terms of clinical symptoms and signs by a fixed attending neurologist, which were divided into the following three types according to duration and severity: nonneurological dysfunction (NND), no obvious neurological damage manifestation on clinical observation; transient neurological dysfunction (TND), postoperative neurological deficit that include conscious disturbances (including coma, lethargy, paralysis, etc.), sensory or motor impairment, and complete disappearance of all neurological damage symptoms before discharge; permanent neurological dysfunction (PND), postoperative neurological deficits that include new onset of coma, sensory or motor impairment, and any neurological damage symptoms that did not completely disappear before discharge.

### 2.4 Statistical analysis

All data were statistically analyzed using SPSS 22.0 (International Business Machines Corporation, USA) software. Multiple sets of independent quantitative data were analyzed using analysis of variance (ANOVA) and LSD‐*t* multiple test, and the catagorical data were analyzed using chi‐square test. The variables were screened by single factor analysis, and *p* < 0.1 was set as the significant difference. Logistic regression analysis was used to analyze the correlation between the selected variables and the dependent variables. *p* < 0.05 was set as the significant difference. The best cut‐off value was determined using ROC curve.

The statistics related to the rSO_2_ variables were defined as follows:

rSO_2_ basal value (%): stable rSO_2_ reading that was observed 2 min at each side of forehead after induction of anesthesia.

Relative rSO_2_ (ΔrSO_2_, %) = 100% × rSO_2_ at each time point/rSO_2_ basal value.

The lowest value of rSO_2_: the lowest value of rSO_2_ observed in each time period and lasted for more than 3 min.

Relative rSO_2_ lowest value (ΔrSO_2_min, %) = 100% × rSO_2_ lowest value/rSO_2_ basal value.

## 3 RESULTS

There were 98 patients enrolled in our study, the average age was 50.41 ± 11.38 years old, and the mean BMI was 24.31 ± 3.35. The mean operative time was 340.03 ± 66.90 min, the mean SACP time was 38.18 ± 12.05 min, the mean CPB time was 167.39 ± 34.63 min, and the mean hemostasis time (starting from infusion of protamine to the beginning of the chest closure) was 111.96 ± 28.88 min. The average reading of bilateral basal rSO_2_ was 66.21 ± 9.87%, and the average intraoperative ΔrSO_2_min was 78.74 ± 14.11%. Postoperative all‐cause mortality and neurological prognosis were shown in Table 1. Forty‐two patients experienced postoperative neurological complications (including sensory, motor and/or disturbance of consciousness), among which 29 cases (29.6%) were TND, and 13 cases (13.3%) were PND.

**Table 1:** Overall neurologic outcomes

Outcome	No. (%)
All‐cause mortality	15 (15.3)
Neurological dysfunction	
NND	56 (57.1)
TND	29 (29.6)
PND	13 (13.3)
Motor or sensory disorder	
Temporary disorder	7 (7.1)
Permanent disorder	8 (8.2)
Consciousness disorder	
Temporary disorder	33 (33.7)
Permanent disorder	3 (3.1)
Consciousness outcome	
Clear consciousness	62 (63.3)
Delirium	21 (21.4)
Lethargy	3 (3.1)
Coma	12 (12.2)

Abbreviations: NND, nonneurological dysfunction; TND, transient neurological dysfunction; PND, permanent neurological dysfunction.

Univariate analysis was performed using one‐way ANOVA and LSD‐t multiple test for continuous variables, and chi‐square test for categorical variables between the NND group, TND group and PND group. The results showed that the preoperative platelet counts were lower in the NND group than in the other two groups. The proportion of males in the TND group was higher than that in the NND group. The hemostasis time in the PND group was longer than that in the NND group. The age of the PND group was higher than the other two groups. The ΔrSO_2_min indexes in the PND group during the whole operation, the SACP, the CPB and the hemostasis were lower than that in the other groups (*p* < 0.1, Table 2).

**Table 2:** Preoperative demographics, patient‐related factors, laboratory variables and characterizing intraoperative factors in patients with NND, TND or PND^a^

Variable	NND (*n* = 56)	TND (*n* = 29)	PND (*n* = 13)	*p‐*Value
Male	38 (67.9%)	26 (89.7%)	10 (76.9%)	0.085
Age (years)	50.48 ± 10.6	47.52 ± 12.83	56.54 ± 9.32	0.058
BMI (kg/m^2^)	24.42 ± 3.54	24.27 ± 3.16	23.95 ± 3.07	0.902
Marfan Syndrome	8 (14.3%)	3 (10.3%)	0 (0.0%)	0.165
Hypertension	40 (71.4%)	22 (75.9%)	11 (84.6%)	0.605
Diabetes	6 (10.7%)	5 (17.2%)	2 (15.4%)	0.686
LVEF (%)	61.13 ± 15.86	60.38 ± 18.80	59.69 ± 18.49	0.956
NT‐proBNP(pg/ml)	437.82 ± 715.29	544.10 ± 690.21	465.16 ± 667.79	0.803
Hb (g/L)	124.02 ± 16.39	129.24 ± 17.77	130.15 ± 16.39	0.277
WBC (×10^9^/L)	10.76 ± 14.59	9.99 ± 4.66	12.52 ± 4.10	0.804
PLT(×10^9^/L)	231.68 ± 95.14	197.90 ± 58.04	158.62 ± 65.01	0.011
Alb (g/L)	37.54 ± 6.28	40.38 ± 5.17	39.38 ± 6.74	0.112
ALT (U/L)	33.46 ± 36.58	39.41 ± 79.87	30.17 ± 23.83	0.838
AST (U/L)	29.88 ± 17.80	35.03 ± 71.45	54.00 ± 64.66	0.274
BUN (mmol/L)	5.90 ± 1.87	6.57 ± 2.29	6.42 ± 1.67	0.312
Scr (μmol/L)	83.23 ± 27.24	90.93 ± 43.77	78.77 ± 25.83	0.458
INR	1.21 ± 1.66	0.98 ± 0.21	1.00 ± 0.32	0.691
Mean rSO_2_ basal value	64.34 ± 8.84	67.05 ± 12.01	62.23 ± 8.55	0.295
ΔrSO_2_min (%)				
Throughout operation	79.37 ± 12.03	82.62 ± 12.88	67.38 ± 19.51	0.004
During SACP	90.00 ± 17.77	94.99 ± 15.61	76.95 ± 19.71	0.010
Throughout CPB	80.76 ± 12.67	84.79 ± 12.62	70.67 ± 18.49	0.009
During hemostasis	89.53 ± 12.47	89.96 ± 20.23	78.89 ± 16.78	0.086
Intraoperative factors				
LNT in DHCA(°C)	18.79 ± 1.34	18.28 ± 1.14	19.02 ± 1.17	0.122
Surgery time (min)	333.46 ± 67.97	343.48 ± 67.70	360.62 ± 60.16	0.401
SACP time (min)	36.86 ± 11.71	39 ± 13.54	42.08 ± 9.55	0.342
CPB time (min)	165.38 ± 34.54	172.31 ± 39.35	165.08 ± 22.99	0.664
Hemostasis time (min)	107.32 ± 29.15	114.86 ± 26.01	126.23 ± 29.71	0.083

Abbreviations: NND, nonneurological dysfunction; TND, transient neurological dysfunction; PND, permanent neurological dysfunction; BMI, body mass index; LVEF, left ventricular ejection fraction; NT‐proBNP, N‐terminal pro‐B‐type natriuretic peptide; Hb, hemoglobin; WBC, white blood cell count; Alb, albumin; ALT, alanine aminotransferase; AST, aspartate aminotransferase; BUN, blood urea nitrogen; Scr, serum creatinine; INR, international normalized ratio; SACP, selective antegrade cerebral perfusion; CPB, cardiopulmonary bypass; LNT, lowest nasopharyngeal temperature; DHCA, deep hypothermic circulatory arrest. Values are presented as the mean ± *SD* or number (percentage).

a LSD test was used for post hoc analysis.

Multiple logistic regression was further used to analyze the effects of gender, age, preoperative platelet count, hemostasis time, intraoperative ΔrSO_2_min, ΔrSO_2_min in SACP, ΔrSO_2_min in CPB, and ΔrSO_2_min during hemostasis on NND, TND and PND (Tables 3 and 4). The results showed that age, preoperative platelet count, hemostasis time and ΔrSO_2_min were risk factors for postoperative PND. Compared with NND, patients with preoperative low platelet count, prolonged hemostasis time and decreased ΔrSO_2_min in each time period were more likely to experience postoperative PND. Compared with TND, patients with advanced age and decreased ΔrSO_2_min in each time period were more likely to experience postoperative PND. Each factor had no effect on the difference between the TND and NND groups.

**Table 3:** Logistic regression model explaining probability difference between PND and NND

Model by duration	Parameter	OR value	95% CI	*p*‐Value
Whole operation	Age	1.099	(1.005–1.202)	0.039
	ΔrSO_2_min	0.925	(0.871–0.982)	0.011
	Platelet	0.988	(0.978–0.999)	0.034
	Hemostasis time	1.043	(1.009–1.078)	0.014
	Female	0.245	(0.039–1.554)	0.135
SACP	Age	1.094	(0.998–1.199)	0.055
	ΔrSO_2_min	0.927	(0.872–0.986)	0.015
	Platelet	0.984	(0.973–0.996)	0.009
	Hemostasis time	1.047	(1.010–1.086)	0.012
	Female	0.202	(0.029–1.429)	0.109
CPB	Age	1.088	(1.000–1.185)	0.050
	ΔrSO_2_min	0.936	(0.882–0.993)	0.028
	Platelet	0.988	(0.977–0.999)	0.025
	Hemostasis time	1.038	(1.005–1.071)	0.022
	Female	0.248	(0.038–1.615)	0.145
Hemostasis	Age	1.075	(0.991–1.165)	0.082
	ΔrSO_2_min	0.943	(0.894–0.996)	0.034
	Platelet	0.988	(0.977–0.999)	0.029
	Hemostasis time	1.042	(1.010–1.076)	0.011
	Female	0.325	(0.054–1.938)	0.217

Abbreviations: PND, permanent neurological dysfunction; NND, nonneurological dysfunction; OR, odds ratio; CI, confidence interval; SACP, selective antegrade cerebral perfusion; CPB, cardiopulmonary bypass.

**Table 4:** Logistic regression model explaining probability difference between PND and TND

Model by duration	Parameter	OR value	95% CI	*p*‐Value
Whole operation	Age	1.127	(1.027–1.237)	0.012
	ΔrSO_2_min	0.901	(0.844–0.963)	0.002
	Platelet	0.994	(0.983–1.006)	0.311
	Hemostasis time	1.033	(0.998–1.068)	0.066
	Female	0.891	(0.107–7.403)	0.915
SACP	Age	1.120	(1.019–1.231)	0.019
	ΔrSO_2_min	0.907	(0.850–0.967)	0.003
	Platelet	0.990	(0.978–1.003)	0.117
	Hemostasis time	1.038	(1.000–1.076)	0.049
	Female	0.761	(0.082–7.028)	0.810
CPB	Age	1.116	(1.022–1.219)	0.015
	ΔrSO_2_min	0.906	(0.849–0.968)	0.003
	Platelet	0.994	(0.982–1.005)	0.286
	Hemostasis time	1.028	(0.995–1.063)	0.093
	Female	0.890	(0.105–7.583)	0.915
Hemostasis	Age	1.092	(1.005–1.187)	0.039
	ΔrSO_2_min	0.939	(0.888–0.993)	0.026
	Platelet	0.993	(0.982–1.005)	0.249
	Hemostasis time	1.031	(0.998–1.064)	0.067
	Female	1.191	(0.157–9.046)	0.866

Abbreviations: PND, permanent neurological dysfunction; TND, transient neurological dysfunction; OR, odds ratio; CI, confidence interval; SACP, selective antegrade cerebral perfusion; CPB, cardiopulmonary bypass.

**Figure 1:**
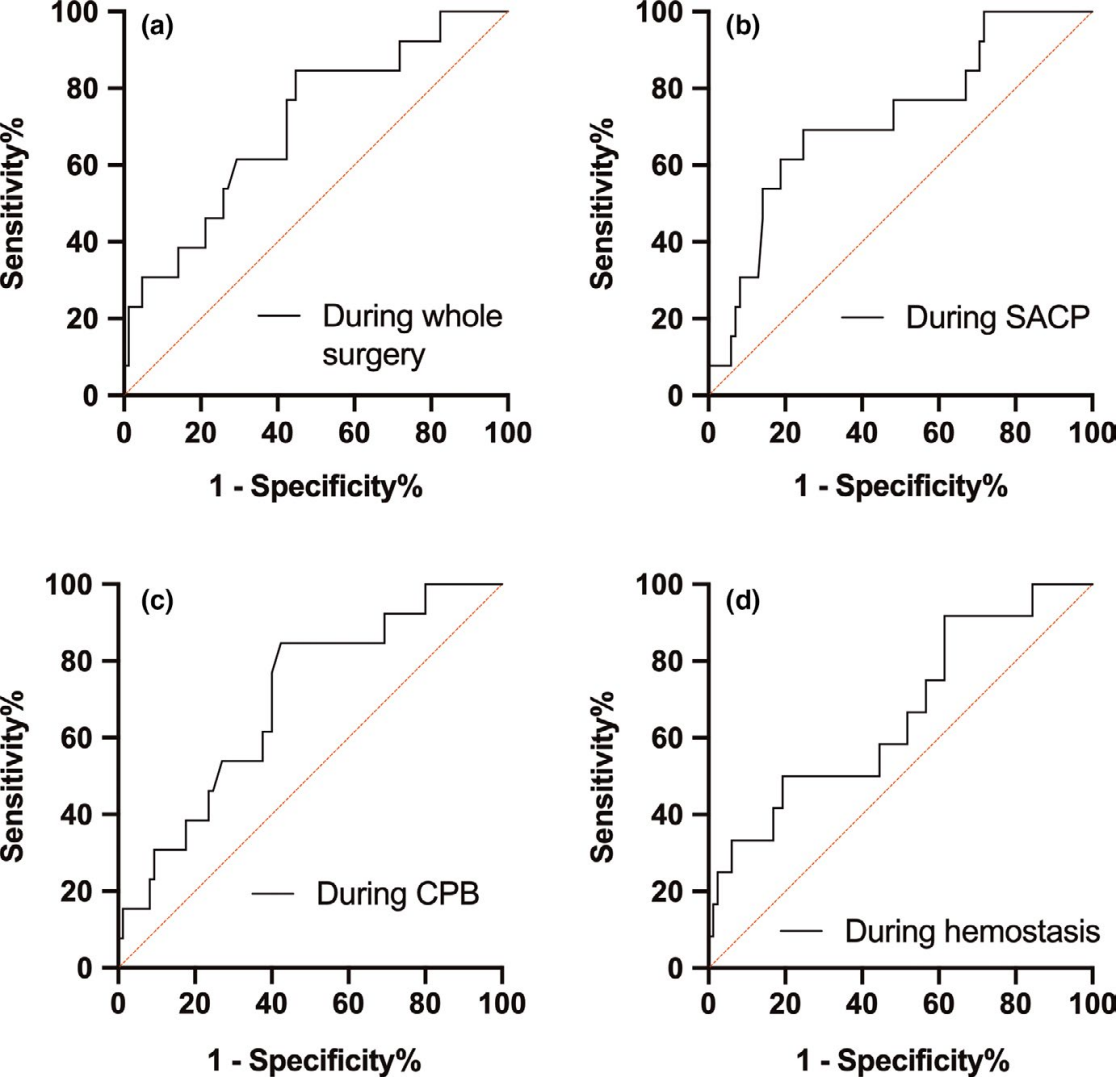
ROC curves on PND for lowest rSO_2_ relative to baseline during the whole surgery (a), during SACP (b), during CPB (c), and during the hemostasis period (d)

The ROC curve was used to measure the ΔrSO_2_min in each time period to predict the cut‐off value of PND occurring after total arch replacement. The results suggested that the optimal cut‐off value of ΔrSO_2_min for intraoperative PND was 79.7%, the area under the curve was 0.708 (95% confidence interval = 0.557–0.858), *p* = 0.016; the optimal cut‐off value of ΔrSO_2_min in SACP was 81.6%, the area under the curve was 0.720 (95% confidence interval was 0.570–0.870), *p* = 0.011. The optimal cut‐off value of ΔrSO_2_min in CPB was 80.8%, the area under the curve was 0.697 (95% confidence interval is 0.554–0.840), *p* = 0.023 (Table 5; Figure 1).

**Table 5:** Diagnostic performance indices of lowest relative rSO_2 _during each period in predicting PND

Parameter	ΔrSO_2_min during			
	Whole surgery	SACP	CPB	Hemostasis
Cut‐off value (%)	79.7	81.6	80.8	78.6
AUC	0.708	0.720	0.697	0.662
95%CI	(0.557–0.858)	(0.570–0.870)	(0.554–0.840)	(0.492–0.831)
Sensitivity (%)	55.3	75.3	57.6	80.7
Specificity (%)	84.6	69.2	84.6	50.0
*p*‐Value	0.016	0.011	0.023	0.071

Abbreviations: AUC, area under curve; CI, confidence interval; SACP, selective antegrade cerebral perfusion; CPB, cardiopulmonary bypass.

## 4 DISCUSSION

Total aortic arch replacement is technically difficult, and it directly involves the anastomosis of blood vessels in both head and neck. Central nervous system injury and brain function protection are always the focus of research in various cooperative disciplines. During the operation, when the autologous blood vessel and artificial blood vessel are anastomosed at the beginning of the descending aorta, the systemic blood supply must be suspended to expose the surgical field. Even with cerebral blood perfusion combined with deep hypothermia and local vascular (often radial or innominate) cannula, its neurological complications are still much higher than other cardiac surgeries (Shelstad, Reeves, Yamanaka, & Reece, 2016). In animal models, it has been found that DHCA can significantly reduce mitochondrial respiratory chain complex 1 and increase reactive oxygen species. Cerebral ischemia indexes (lactic acid/pyruvate ratio) which increase during following subsequent warming may be one of the mechanisms leading to impaired brain function (Mavroudis et al., 2018). Stanford type A aortic dissection is the most common disease requiring total arch replacement that is usually complicated by hypertension, atherosclerosis or Marfan syndrome. Clinically, these conditions are critical and always need emergent operations (Settepani, Cappai, Basciu, Barbone, & Tarelli, 2016). The mortality rate of emergency total arch replacement surgery is 6.5%–16.3% (Eusanio et al., 2004; Minakawa et al., 2010; Okita et al., 2013; Shimizu, Matayoshi, Morita, Ueda, & Yozu, 2013), and incidence of PND or stroke was 10%–18% (Apostolidou et al., 2012; Olsson & Thelin, 2006).

In this study, patients with acute A‐type aortic dissection emergency total arch replacement were enrolled, aiming at finding a predictive effect on the prognosis of the nervous system during the perioperative period, in order to improve the prognosis. After collecting and analyzing a number of preoperative and intraoperative data, it showed decreased ΔrSO_2_min, advanced age, low platelet count, and prolonged hemostasis time were associated with increased postoperative PND incidence. This study failed to find the differences in the factors between NND and TND, that is to say, the predictive factors for TND could not be found; this might be because of limited rSO_2_ data (such as low duration or area under the curve, etc.) or screening factors that were not widely classified. However, the symptoms of TND could disappear before discharge, and the occurrence of PND has a greater impact than TND on the quality of life of patients after surgery, so the predictive factors should also be more valued. In our study, it was found that the optimal cut‐off value of ΔrSO_2_min for predicting PND in all operation steps was about 80% of the baseline value (Table 5), AUC was about 0.7, and the fit was good, which may be related to the PND sample size that was only 13 cases. Olsson and Thelin (2006) conducted a similar study, which included and observed changes in rSO_2_ during SACP in 46 patients undergoing aortic arch surgery, suggesting that the range of 76%–86% of rSO_2_ in the SACP process has a suggestive effect on postoperative stroke. Schön et al. (2009) suggested that the incidence of postoperative stroke was higher in the DHCA + arch surgery with rSO_2_ <80% baseline value than in the control group. Another study (Fischer et al., 2011) reported that an rSO_2_ reading <65% was associated with severe complications after arch surgery, with a stroke rate of 3/30 (10%). Combining the results of this study and previous studies, it is believed that intraoperative ΔrSO_2_min decline is associated with postoperative central nervous system complications. Compared with previous studies, this study focused on type A dissection emergency surgery, and stratified analysis results based on neurological prognosis severity: ΔrSO_2_min <80% is more strongly correlated with PND than TND, and it is suggested that monitoring ΔrSO_2_min in such surgeries can be used to predict severe neurological complications.

It is found in this study that advanced age is associated with postoperative PND, which is clearly associated with decreased functional reserve in older patients. Also, preoperative low platelet count and prolonged hemostasis time are associated with PND. Interestingly, platelet count in the PND group (158.62 ± 65.01 × 10^9^/L) was not below normal but significantly lower than in the TND and NND groups. This may be due to the fact that preoperative basal vasculopathy was severe in patients with PND, leading to platelet activation and consumption. On the other hand, CPB itself causes coagulation factor dilution, blood clot formation disorders, fibrinogen dysfunction, platelet dysfunction, platelet destruction, or fibrinolysis, which is one of the important reasons for the difficulty in stopping bleeding in the surgical field post‐CPB (Ranucci, 2015). Low temperature during DHCA further inhibits platelet function and aggravates coagulation factor dilution by prolonging CPB time (Hanna et al., 2016). Therefore, patients with normal preoperative coagulation function often experience coagulopathy during hemostasis after CPB. The controlled hypotension used in combination with surgical hemostasis reduce systemic organ perfusion including brain tissue, and low‐level brain tissue oxygen supply for a long time shows low levels of ΔrSO_2_, which may be the one of the reasons for postoperative PND. Perhaps the use of point of care platelet counts and functional monitoring in such surgeries can help early detection and correction platelet count or function disorders after CPB to decrease the time of hemostasis and controlled hypotension.

The study still has the following shortcomings. It was a single‐center retrospective study, and data collection was inevitably biased. In addition, the intraoperative rSO_2_ data collected were characteristic rather than continuous, which might be inaccurate with the real situation and mask some actual correlations, requiring further studies.

In summary, low ΔrSO_2_min during all stages of surgery (the whole surgery, CPB, SACP, and hemostasis), advanced age, preoperative low platelet count, and prolonged hemostasis time are all predictors of PND after thoracic aortic total arch replacement. For elderly patients, positive correction of the platelet count perioperatively, positive correction and maintenance of ΔrSO_2_ higher than 80% of the baseline value intraoperatively may effectively reduce postoperative PND and improve postoperative quality of life.

## AUTHOR CONTRIBUTIONS

YY, YL, and GK formulated the design of the study, carried out the execution and analysis of the study, and drafted the manuscript. LJ, LX, HW, and YR were involved in data collection.

## Data Availability

All original data will be available when you contact the correspondence author.
